# 2796. A Decade of *Rhodococcus* spp. Infections: Microbiology, Antimicrobial Susceptibility, and Clinical Experience

**DOI:** 10.1093/ofid/ofad500.2407

**Published:** 2023-11-27

**Authors:** Nischal Ranganath, Maria Alejandra Mendoza, Ryan W W Stevens, Nancy Wengenack, Aditya Shah

**Affiliations:** Mayo Clinic, Rochester, Minnesota; Mayo Clinic, Rochester, Minnesota; Mayo Clinic, Rochester, Minnesota; Mayo Clinic, Rochester, Minnesota; Mayo Clinic, Rochester, Minnesota

## Abstract

**Background:**

*Rhodococcus* spp. are a zoonotic, Gram-positive coccobacilli that cause pulmonary and disseminated disease in immunocompromised patients. *Rhodococcus* infection is associated with high morbidity and mortality and treatment can be challenging due to intrinsic resistance to several antibiotics. There is limited data on antibiotic susceptibility patterns and clinical characteristics of infection in humans.

**Methods:**

We retrospectively evaluated the microbiology and antibiotic susceptibility testing (AST) of *Rhodococcus* isolates submitted to our medical reference laboratory between June 2012 and 2022. Identification (ID) was performed by Sanger 16S rRNA gene sequencing or MALDI-TOF mass spectrometry. AST was performed by microbroth dilution. We also reviewed the clinical characteristics, management, and outcomes of patients managed at our local academic medical center.

**Results:**

Over a 10-year period, 322 isolates of *Rhodococcus* spp. were identified from blood (50%), pulmonary (26%), and bone/joint (12%) sources. *R equi/hoagii* and *R corynebacteriodes* were the most frequently isolated species, with 19% of isolates only identified to genus level (Figure 1). 199 isolates were evaluated for AST, with high rates of resistance to cephalosporins and aminoglycosides. Optimal antibiotics based on AST included imipenem, vancomycin, linezolid, clarithromycin, and TMP-SMX. Discordance in susceptibility was noted between ciprofloxacin (62%) and moxifloxacin (94%) (Table 1). Ten clinical cases of *Rhodococcus* infection were identified. Eight patients were immunocompromised with 6 having pulmonary and 3 with extra-pulmonary disease. Significant variability in choice and duration of therapy was noted, but all patients had positive treatment response with minimal complications (Table 2).

**Figure:**
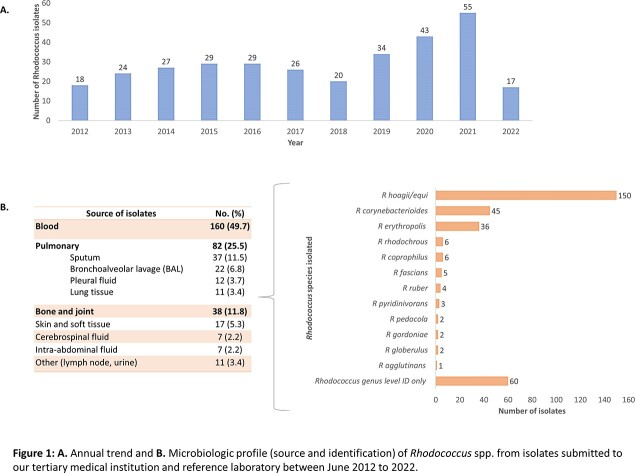

**Figure:**
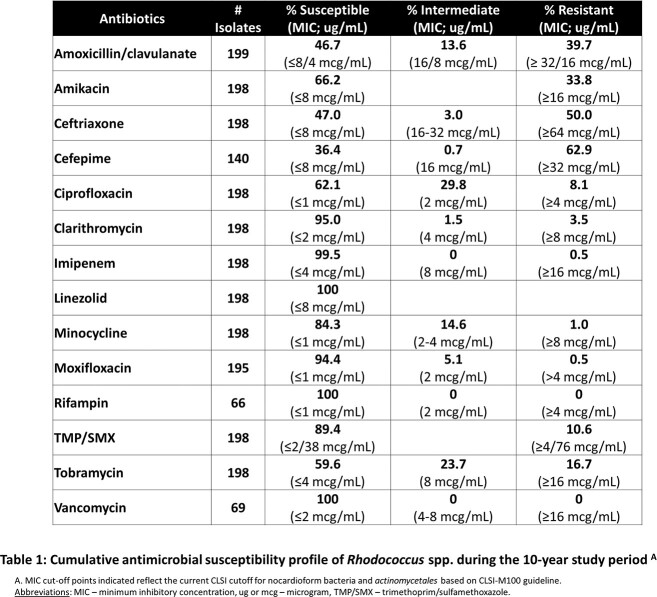

**Figure:**
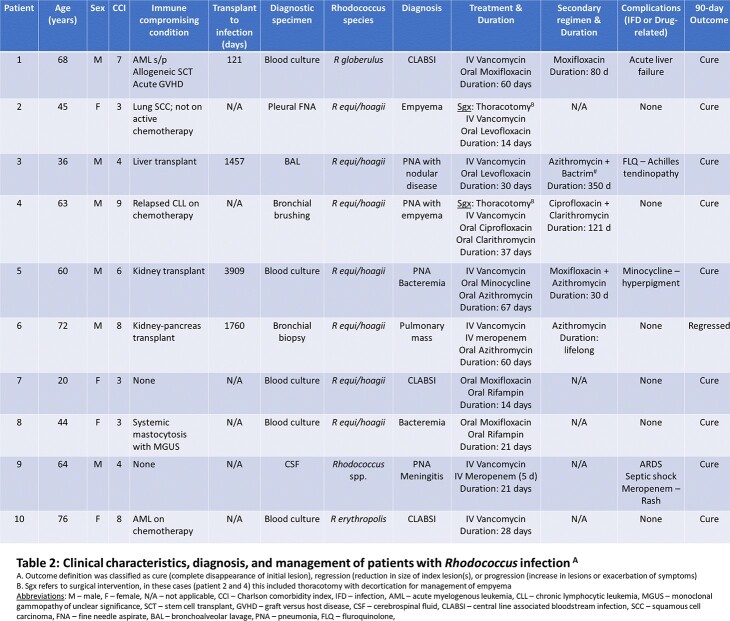

**Conclusion:**

This study demonstrates high rates of resistance among *Rhodococcus* isolates with need for accurate ID and AST to guide effective treatment of infection among immunocompromised patients.

**Disclosures:**

**Nancy Wengenack, PhD**, Mayo Clinic Laboratories: Employee

